# Determinants of the Bovine Leukemia Virus Envelope Glycoproteins Involved in Infectivity, Replication and Pathogenesis

**DOI:** 10.3390/v8040088

**Published:** 2016-03-24

**Authors:** Alix de Brogniez, Jan Mast, Luc Willems

**Affiliations:** 1 Molecular and Cellular Epigenetics (GIGA) and Molecular Biology (Gembloux Agro-Bio Tech), University of Liège (ULg), 4000 Liège, Belgium; 2 Veterinary and Agrochemical Research Center CODA-CERVA, 1180 Brussels, Belgium; jan.mast@coda-cerva.be

**Keywords:** retroviruses, viral entry, envelope, glycoprotein, J0101

## Abstract

Interaction of viral envelope proteins with host cell membranes has been extensively investigated in a number of systems. However, the biological relevance of these interactions *in vivo* has been hampered by the absence of adequate animal models. Reverse genetics using the bovine leukemia virus (BLV) genome highlighted important functional domains of the envelope protein involved in the viral life cycle. For example, immunoreceptor tyrosine-based activation motifs (ITAM) of the envelope transmembrane protein (TM) are essential determinants of infection. Although cell fusion directed by the aminoterminal end of TM is postulated to be essential, some proviruses expressing fusion-deficient envelope proteins unexpectedly replicate at wild-type levels. Surprisingly also, a conserved *N*-linked glycosylation site of the extracellular envelope protein (SU) inhibits cell-to-cell transmission suggesting that infectious potential has been limited during evolution. In this review, we summarize the knowledge pertaining to the BLV envelope protein in the context of viral infection, replication and pathogenesis.

## 1. Introduction

**Figure 1:**
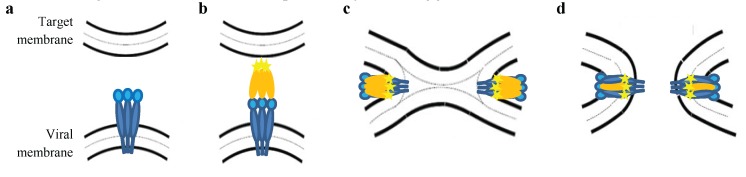
Membrane fusion of bovine leukemia virus (BLV) envelope protein based on the Murine Leukemia virus fusion model. (**a**) Fusion incompentent state of the envelope complex formed by the receptor-binding (surface protein (SU), gp51 in light blue) and the fusion (transmembrane protein (TM), gp30 in dark blue) subunits; (**b**) After receptor binding, a conformational change exposes the fusion peptide (yellow star) to the target cell membrane; (**c**) Insertion of the fusion peptide into the lipid bilayer mediates formation of a hemifusion diaphragm and blending of viral and cellular lipids (gray dots); (**d**) Fusion structure after refolding. In this state the fusion peptide and the TM are anchored into the same membrane in an anti-parallel conformation.

Infection by enveloped viruses requires the successive action of two distinct glycoproteins (surface protein (SU) and transmembrane protein (TM)) anchored into the lipid bilayer surrounding the virion. Both glycoproteins derive from the post-translational cleavage of a precursor encoded by the *env* gene and are associated by a unique disulfide bond linking cysteine residues of SU CXXC and TM CX_6_CC motifs (where X represents a variable residue) [1,2,3,4]. Although the mechanisms are still unknown, the most widely accepted entry process for retroviruses follows the Murine Leukemia virus fusion model (Figure 1). The SU receptor-binding domain (RBD) interacts with specific receptor(s) on the target cell. This interaction induces a conformational change that initiates the fusion process directed by TM. Before SU-receptor interaction, the TM protein is maintained in a fusogenic-inactive metastable state in which the fusion peptide is hidden. After receptor binding, the disulfide bridge linking SU and TM [4] is disrupted allowing refolding of TM into a fusogenic conformation. The fusion peptide located at the NH_2_ terminal part of TM destabilizes the cell membrane resulting in the opening of the lipid bilayer and release of the viral nucleocapsid into the host cell cytoplasm. This process requires the formation of a six-helix coiled coil bundle that brings the viral and target membrane in close proximity and triggers membrane fusion [2].

This review focuses on the functional domains of the BLV envelope glycoproteins and their impact on the viral life cycle. BLV is a deltaretrovirus that induces hematological diseases in ruminants. Although natural hosts are cattle, zebu and water buffalo, BLV can also be experimentally transmitted to sheep (see [5,6,7] for recent reviews). The advantage of the ovine species is that disease is faster and more frequent than in cattle [8,9,10] allowing to characterize the physiopathology of leukemia-lymphoma. BLV encodes an oncogenic protein called Tax able to transform primary cells and microRNAs that affect host cell gene expression [7,11,12,13]. Infection is mediated by the interaction of gp51 with a still unknown receptor.

## 2. The SU Glycoprotein

Since viral particles are very unstable, the main route of viral infection is believed to involve cell-associated virus [14]. In this process, an infected B-lymphocyte expressing envelope protein at the external membrane can undergo fusion with a new target cell. Distinct structural domains of SU have been identified.

### 2.1. SU Interacts with Zn

**Figure 2:**
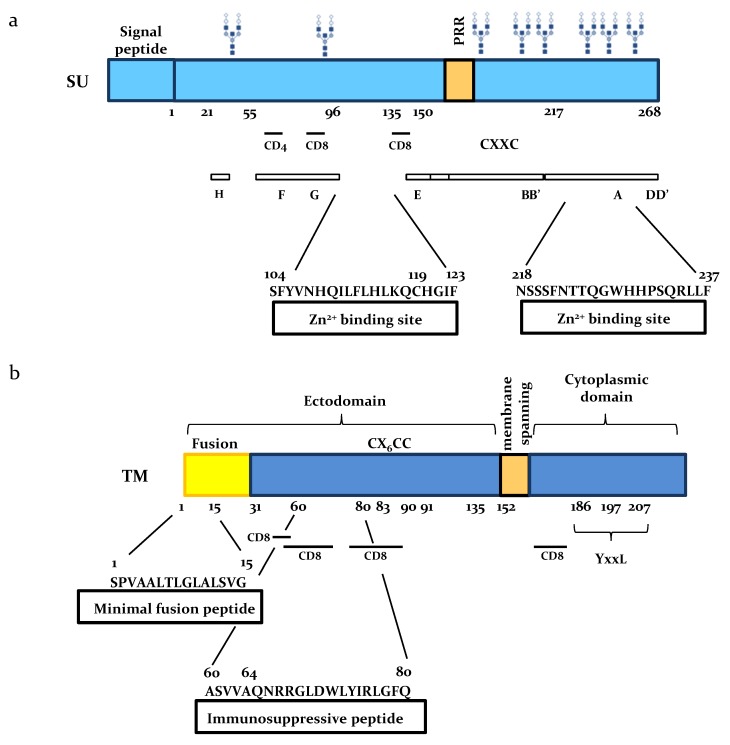
Schematic representation of the SU and TM envelope proteins. (**a**) Major domains of BLV SU are indicated: potential *N*-glycosylation sites (glycan trees), antigenic peptides targeted by monoclonal antibodies (A-H), CD4 epitope at amino acid 61–70 and CD8 epitopes respectively located at position 73–92 and 131–140 in the SU protein and at amino acid 40–59; 50–69; 70–89 and 150–169 in the TM part, a proline-rich region (PRR) and two zinc-binding regions (Zn^2+^). Numbers are amino acid coordinates of SU after signal peptide cleavage (green); (**b**) The TM protein has two hydrophobic regions: a fusion peptide (yellow) and a membrane-spanning domain (orange) that anchors the SU-TM complex into lipid bilayers. Other TM domains are: the immunosuppressive peptide, immunoreceptor tyrosine-based (YxxL) activation motifs (ITAM) and cysteines (C) involved in disulfide bonds between SU and TM.

The ability of SU proteins to interact with specific ligands was investigated by affinity chromatography [15]. These experiments revealed that SU amino acids 104–123 and 218–237 interact with Zn^2+^ ions using cysteine and histidine residues as structural binding sites (Figure 2). Hydrophobic cluster analysis (HCA) and 3-D structure of the Friend murine leukemia virus (Fr-MLV) RBD positioned the first zinc-binding peptide on the opposite site of the potential receptor binding site proposed for the BLV SU, suggesting that Zn^2+^ ions could mediate interaction either with the rest of the envelope protein or with partners different from the receptor. Reverse genetics showed that the integrity of the cysteines was essential for fusogenic activity of the SU-TM complex.

### 2.2. BLV SU is Immunogenic

BLV SU is a major target of anti-viral immunity, as indicated by the rapid emergence of neutralizing antibodies after viral inoculation. SU has at least 8 antigenic sites arbitrarily named A to H among which three correspond to conformational and neutralizing epitopes (Figure 2). Monoclonal antibodies directed against epitope H are able to completely inhibit cell fusion in culture. Anti-F and anti-G monoclonals reduce less efficiently the ability to form syncytia. Among all known BLV strains, the simultaneous loss of these three epitopes has never been reported suggesting their important role in the viral life cycle [16,17]. Besides humoral immunity, SU also stimulates a T-cell response as indicated by the presence of helper CD4+ and cytotoxic T-lymphocyte (CTL) CD8+ epitopes [18] (Figure 2). In contrast to SU, BLV TM is very poorly immunogenic.

### 2.3. Role of SU N-Linked Glycosylation

Retroviral envelope glycosylation mediates virion attachment to cell membranes and fusion [19,20,21,22]. On the other hand, envelope associated glycans also act as a shield that confers resistance to neutralizing antibodies [21,23]. As other retroviral envelope proteins, the BLV 51 kDa SU protein is highly glycosylated since the molecular weight of its peptidic backbone in absence of any glycan is only 30.5 kDa. SU has 8 Asn-X-Ser/Thr consensus *N*-glycosylation sites, X being any amino acid except a proline (Figure 2). The role of these glycosylation sites in viral replication and pathogenesis was recently demonstrated by reverse genetics. Simultaneous mutation of the 8 sites abrogated infectivity *in vivo* consistent with a role in the viral persistence or replication [24]. In contrast, single mutations of the glycosylation sites were almost silent except one at asparagine N230. This particular N230 mutation stabilized the SU protein and increased cell-to-cell infection *in vitro*. Unexpectedly, a provirus carrying the N230 mutation replicated faster than wild-type and was hyper-pathogenic *in vivo*. To our knowledge, this particular mutant is the only example identified so far among delta-retroviruses that is more replication competent and pathogenic than the wild-type strain. 

## 3. Functional Domains of the TM Subunit

The TM subunit of the BLV envelope complex contains distinct domains: an aminoterminal extracellular ectodomain, a hydrophobic membrane-spanning region that anchors the envelope protein into lipid bilayers and a carboxyterminal cytoplasmic tail (Figure 2).

### 3.1. The Ectodomain

The extracellular domain of TM contains two well-defined peptides involved in cell fusion and immunosuppression (Figure 2). Upon refolding of the latent form of TM, the fusion peptide is exposed to the target cell membrane and inserts obliquely into lipid bilayers [25,26]. This peptide is rich in small amino acids such as alanine and glycine that create a hydrophobicity gradient, destabilize the cell membrane and catalyze fusion [27]. The minimal fusion peptide (*i.e.*, the shortest peptide with an optimal tilted angle in the membrane) is composed of 15 amino acids (Figure 2). The TM immunosuppressive region is also involved in the cell fusion process because conservative mutations introduced at amino acid positions 60 and 64 completely abrogate syncytium formation [26]. Surprisingly, these mutations do not abolish infectivity nor limit viral propagation *in vivo* as these mutants can replicate at wild type levels and induce leukemia in the ovine model [26].

### 3.2. The Cytoplasmic Tail

The cytoplasmic tail of BLV TM is characterized by the presence of ITAM motifs characterized by the consensus YxxL (where x represents a variable residue) and located at positions 186, 197 and 207 according to the reference [26] (Figure 2). These ITAM motifs are able to transmit membrane-stimulated signals and may thereby modulate activation of infected B cells [28]. Two tyrosine residues located at positions 186 and 197 are required for viral infectivity and viral propagation *in vivo* [29]. Replacement of these tyrosine residues affects the efficiency of viral entry into cells as well as incorporation of SU and TM proteins into the new viral particles [30].

Although the mechanisms are still unknown, the high degree of conservation of the ITAM motifs in all known BLV strains suggests an important role in the viral life cycle [19,29,31,32].

## 4. Conclusions

The BLV SU and TM envelope proteins are key mediators of virion interaction with cell membranes and lipid bilayer fusion. In this review, we provided updated information on the envelope domains required for viral infectivity, persistence and pathogenesis. This knowledge highlighted potential threats associated with outbreaks of a hyperpathogenic strain but also opened new prospects for the development of vaccines [5]. In a perspective of comparative virology, unraveling the mechanisms of persistence, replication and pathogenesis in the BLV model may also be informative to better understand the physiopathology of a closely related deltaretrovirus, Human T-cell Leukemia virus 1 (HTLV-1), that induces leukemia in humans [6].
